# Total, Patient and System Diagnostic Delays for Pulmonary Bacilliferous Tuberculosis in the Six Diagnostic and Treatment Centers in the Five Health Districts of the Central Region, Burkina Faso, 2018

**DOI:** 10.1007/s44197-021-00027-z

**Published:** 2022-01-03

**Authors:** Pauline Kiswendsida Yanogo, Clarisse Balima, Nicolas Meda

**Affiliations:** 1 Burkina Field Epidemiology Training Program, Faculty of Medicine, Joseph KI-ZERBO University, 06 BP 9268, Ouagadougou 06, Burkina Faso; 2 Department of Public Health, Faculty of Medicine, Joseph KI-ZERBO University, Ouagadougou, Burkina Faso; 3 Universitary Hospital Yalagdo, Ministry of Health, Ouagadougou, Burkina Faso

**Keywords:** Pulmonary tuberculosis, Diagnostic delays, Ouagadougou, Burkina Faso

## Abstract

**Introduction:**

Long diagnosis delay contributes significantly to the failure to eradicate tuberculosis. The objective of this study was to evaluate the total, patient and system delays in diagnosis of pulmonary bacilliferous in the six tuberculosis Diagnostic and Treatment Centers in the five health districts of the central region in Burkina Faso.

**Methods:**

A descriptive cross-sectional study was conducted among 384 microscopy-positive pulmonary tuberculosis patients in 2018 to address this objective. It concerned the socio-demographic, clinical, microbiological characteristics, and referral location/pathway characteristics of the patients. We then calculated the different delays. The “patient” (time from first symptoms to first consultation), “system” (time from first consultation to first diagnosis) and total (time from first symptoms to diagnosis) median diagnostic delay were estimated.

**Results:**

The median “total”, “patient” and “system” diagnostic times were 37, 21 and 7 days, respectively. Of the 384 patients surveyed, 158 patients or 41.25% of patients had a long total diagnostic delay (> 45 days). The number of patients with a long system diagnostic delay was 125 patients (32.55%; *p* < 0.001) and those with a long patient diagnostic delay were 105 patients (27.34%; *p* < 0.001).

**Conclusion:**

The total diagnosis delay of pulmonary tuberculosis was long for almost half of the patients. Awareness of the signs of tuberculosis among patients and caregivers, and consultation in a health center must be intensified to help considerably reduce these delays.

**Supplementary Information:**

The online version contains supplementary material available at 10.1007/s44197-021-00027-z.

## Introduction

Tuberculosis (TB) is one of the top ten causes of death in the world and the leading cause of death from a single infectious agent, ahead of Human immunodeficiency virus/acquired immunodeficiency syndrome (HIV/AIDS) [1]. The advent of acquired immunodeficiency syndrome has contributed to the increase in the number of cases in the world, where the human immunodeficiency virus is endemic. All organs can be affected, but tuberculosis is localized in the lungs in the vast majority of cases. The World Health Organization (WHO) estimated that 10 million people contracted tuberculosis in 2019 including 25% of cases in Africa with the number of deaths estimated at 1.4 million including 208,000 people living with HIV [2]. In Burkina Faso, 6194 cases of tuberculosis were detected during the year 2018 (Annual activity report).

Tuberculosis was placed in Burkina Faso as a priority health problem in 1995, along with many other diseases. The concern to organize the fight effectively has led to the establishment of a National Program for the fight against Tuberculosis since 1995 and the Directly Observed Treatment Short-Course (DOTS) was adopted in the country. Several actions were undertaken in its strategic plan which covered the period 2018–2022 which aimed to reduce the incidence of tuberculosis in Burkina Faso and the mortality related to this disease. Thus, the incidence of tuberculosis fell from 52 cases per 100,000 inhabitants in 2015 to 49 cases per 100,000 inhabitants in 2018 (Annual activity report). Despite the considerable successes, the challenges in the fight against tuberculosis remain major.

Early detection and diagnosis of tuberculosis is a global priority for TB control efforts and is highlighted in the World Health Organization's TB control strategy [3]. Indeed, rapid identification of cases depends, on the one hand, on the prompt recognition by patients of the symptoms of tuberculosis and the search for appropriate health care, and, on the other hand, on the capacity of the healthcare system to diagnose the disease [4]. Thus, long diagnostic delays can occur either at the patient level or at the health system level. However, the long delays in the diagnosis and consequently in the treatment of TB patients not only increase infectivity in the community, but can also lead to a more advanced disease state which can lead to more complications and expose to a higher risk of death [5]. Studies have estimated long delays in the diagnosis of tuberculosis in high-, middle- and low-income countries which varied significantly for patient diagnosis time of 14 days in France [6]; 59 days in Ghana [5] and up to 199 days in Afghanistan [7]. That linked to the health system varied from 25 days in France [6], 45 days in Ghana [5] and 128 days in Afghanistan [7]. As for the total time to diagnosis, it varied from 68 days, 104 days to 366 days, respectively, in these countries [5–7]. A study conducted in Burkina Faso in 2006 showed a total diagnostic delay of up to 120 days [8]. However, reducing diagnostic times would not only limit the transmission of the bacillus, but also control the disease and above all, allow national tuberculosis programs to “end tuberculosis” by 2035 according to the Sustainable Development Goals [9].

In addition, the last study carried out in Burkina Faso, which is relatively old (2006), is not sufficient to guide the measures to be implemented to reduce the total diagnostic delay in our country. Indeed, the authors of this study did not clearly distinguish between the patient-related diagnostic delay and that related to the health system [8], thus making it necessary to carry out new studies on this subject in Burkina Faso. This study, therefore, as for purpose to evaluate the total, patient and system diagnostic delay in patients with pulmonary bacilliferous tuberculosis in the six tuberculosis Diagnosis and treatment centers (DTC) in the five health districts of the central region of Burkina to help guide the national tuberculosis control program.

## Materials and Methods

### Study Framework

This study took place in the central region of Burkina Faso which contains Ouagadougou, the capital of Burkina Faso. Located in the center of the country; the center has an area of 2805 km ^2^ and an estimated population of 2 966 307 inhabitants in 2019. In terms of health, it is subdivided into five health districts, namely: the district of Baskuy, Boulmiougou, Nongre-Massom, Sig–Noghin and that of Bogodogo. Each of these health districts has a tuberculosis diagnosis and treatment center, respectively, the DTC in Samandin, Pissy, Kossodo, Paul VI and that of Bogodogo for the management of tuberculosis cases. The districts ensure the detection and management of TB cases. It includes: a bacilloscopy unit with a laboratory technician equipped with a microscope, reagents, laboratory consumables, management tools for monitoring activities and a processing unit directed by some agents trained in TB care. The reference level for all DTCs is the National Tuberculosis Control Center which is attached to the National Disease Control Department. It has a vocation as a reference center and pole for research, training and management of specific cases of TB. All the patients were recruited from the five DTCs and from the National Tuberculosis Control Center.

### Type and Period of Study

We conducted a descriptive cross-sectional study on patients diagnosed with baciliferous pulmonary tuberculosis during the year 2018 over a period of six (06) months from July 1 to December 31, 2018 in the six tuberculosis Diagnostic and Treatment Centers in the five health districts of the central region in Burkina Faso.

### Study Population and Non-inclusion Criteria

The study population includes patients at least 15 years of age diagnosed with bacteriologically confirmed pulmonary tuberculosis in the central region and who gave consent to participate in the study. Patients whose clinical condition did not allow answers to the questions were not included. In addition, patients with multidrug-resistant tuberculosis were not included, because multidrug-resistant tuberculosis most often results from previously poorly treated pulmonary TB. This could increase the risk of recall bias to different dates.

### Operational Definitions

Total diagnostic delay was defined as the time interval between the date of onset of signs and the date of diagnosis [5, 10–15]. Employment was defined as an income generator. The diagnostic period for pulmonary tuberculosis is made up of two sub-periods defined as follows:

The patient diagnostic delay which was the time interval between the date of the onset of the signs and the date of the first medical consultation [5, 10, 16–21]. In case of various complaints, we considered the most durable symptom. The system diagnostic delay represented the time interval between the date of the first medical consultation and the date of diagnosis [5, 11, 12, 19, 21–24]. The actual date of diagnosis rarely differed from the date of initiation of treatment; therefore, in medical data, where the date of the diagnosis was omitted, we considered the date of the treatment initiation. Community included life in prison, mine and boarding schools.

### Data Collection

The collection questionnaire has undergone a preliminary test; which made it possible to obtain an adapted collection sheet. The data was collected in the five (5) Diagnostic and Treatment Centers and the National Tuberculosis Control Center during working days. The information was collected from the registers in the respective patient monitoring centers and by means of a questionnaire administered during a face-to-face interview. For each patient, the data were collected by means of an individual sheet providing information on the socio-demographic, clinical and microbiological characteristics, the place of reference/the patient's journey, as well as the various dates allowing the calculation of diagnostic times. Confirmation of information was made for those who did not remember with the help of their relatives and verification of the dates of laboratory examinations. Routine sputum smear microscopy, GeneXpert, and HIV test results were retrieved with patient permission. All these patients were received during this period, some hospitalized and others in ambulatory treatment; investigators returned to the diagnostic and treatment centers each time to interview newly admitted and diagnosed patients.

### Data Management

An input mask was created and the data collected was entered as they were received on a computer and using Epi Info software in version 7.2.2.6. We searched for and integrated missing data and corrected outliers. An anonymous database was set up for the statistical analyses which were done using the STATA software.

### Statistical Analysis

A descriptive analysis was carried out and included the sociodemographic, clinical and microbiological characteristics and the place of reference/patient path of the patients. We then calculated the different delays:

The patient diagnostic delay was calculated by making the difference between the date of the first medical consultation and the date of the onset of signs. The system diagnostic delay was calculated as the difference between the date of diagnosis and the date of first medical consultation. The total diagnostic delay was calculated by making the difference between the date of diagnosis and the date of onset of signs. The median times were then categorized into short and long times according to the following definitions: a threshold of 30 days was considered acceptable for the patient diagnostic delay [5, 10, 16–18]. Thus, a delay of more than 30 days was deemed unacceptable and we categorized this delay as short, ≤ 30 days and long, > 30 days. A threshold of 15 days was applied for the system diagnostic delay [5, 10]. Thus, we split it into a short, ≤ 15 days and a long, > 15 days. For the total diagnostic timeframe, it is short when it is ≤ 45 days and long > 45 days. Results were tabulated as proportions and ratios. Analyses were performed using Stata software.

## Results

### Sociodemographic, Clinical and Biological Characteristics of Bacilliferous Pulmonary Tuberculosis Patients

In total of 384 patients were interviewed during the study period and all gave their voluntary consent to participate in the study. Subjects aged 25 to 44 were predominant (*p* < 0.001).

Among these 384 patients, 287 (74.74%; *p* < 0.001) were predominantly male with a sex ratio of 2.95. The patients resided in urban areas in 336 cases (87.50%) and 48 (12.50%) in rural areas. Among the patients interviewed, 298 (77.60%) had a job and 327 (85.16%) were living in at home and 57 (14.84%) in the community.

They were 40.90% whom had a history of tuberculosis and 16.67% a chronic disease. Cough was the most common symptom reported by our patients (79.70%). HIV serology was positive in 30 patients (7.8% of cases). Bogodogo district had the most patients in our sample (28.9%; *p* < 0.001). The national tuberculosis control center (NTBCC), a reference center, diagnosed 29.68% of patients. Among the 384 patients; the Health and Social Promotion Centers (HSPC) requested microscopic examination of sputum or GeneXpert in 46 patients. Before going to the reference health facility, 35.15% of patients first consulted traditional medicine and 11.20% were self-medicated (*p* < 0.001) (Table 1).

**Table 1:** Sociodemographic, clinical and biological characteristics of bacilliferous pulmonary tuberculosis patients in Ouagadougou, Burkina Faso

Characteristics	Cases (*n* = 384)	Percentage (%)	*p*
Age			< 0.001
[15–24]	94	24.48	
[25–44]	196	51.04	
[45–64]	81	21.09	
≥ 65	13	3.39	
Sex			< 0.001
Female	97	25.26	
Male	287	74.74	
Profession			< 0.001
Employed	298	77.60	
Unemployed	80	20.80	
Health worker	6	1.60	
Residence			*p* < 0.001
Urban	336	87.5	
Rural	48	12.5	
Lodging			*p* < 0.001
Residence	327	85.16	
Community	57	14.84	
Clinical signs			*p* < 0.001
Persistent cough	306	79.7	
Fever	187	48.7	
Sputum	43	11.2	
Weight loss	69	18	
Hemoptysis	9	2.4	
Chest pain	36	9.4	
Nocturnal sweat	22	5,7	
History of tuberculosis			*p* < 0.001
Personal	85	22.1	
Family	69	18.0	
Professional	3	0.8	
No history	227	59.1	
Medical history			*p* < 0.001
HIV	24	6.27	
Other chronic disease	40	10.41	
Without history	320	83.33	
Diagnostic tool			*p* < 0.001
Genexpert	17	4.4	
Microscopy	367	95.6	
HIV serology			*p* < 0.001
Positive	30	7.80	
Negative	354		
Health district			*p* < 0.001
Bogodogo	111	28.9	
Nongr-Massom	64	25.5	
Sig-Noghin	94	24.5	
Boulmiougou	98	16.7	
Baskuy	14	4.4	
Diagnostic center			*p* < 0.001
NTBCC	81	21.10	
MCSA pissy	75	19.53	
MCSA Paul VI	67	17.44	
UH Bogodogo	26	6.78	
MCSA of Kossodo	11	2.87	
UH Yalagdo Ouédraogo	9	2.34	
MC of Samandin	1	0.26	
Referral health facility*			*p* < 0.001
Spontaneous access to diagnostic health center	223	58.07	
HSPC	46	11.98	
MCSA	60	15.63	
Private structure	38	9.89	
UH	17	4.43	
Procedure performed before accessing the referral health facility			*p* < 0.001
Health facility	206	53.65	
Self-medication	43	11.2	
Traditional medicine	135	35.15	

*NTBCC* National Tuberculosis Control Center, *MCSA* Medical center with surgical antenna, *UH* university hospital; *HSPC* Health and social promotion centers

*Referral health facility: the facility which sent the patient to the diagnostic centers. They don’t do TB diagnosis

### Distribution of the Patient, System and Total Diagnostic Delays of Bacilliferous Pulmonary Tuberculosis Patients in Ouagadougou, Burkina Faso

The median total diagnostic delay was 37 days with extremes of 5 and 1100 days and its mean was75.9 days. Diagnosis delay due to the patient was 21 days with extremes of 1 and 720 days and its mean was 50.9 days. Lastly, the median time to diagnosis due to the health system was 7 days with extremes of 1 and 980 days and its mean was 25.03 days (Table 2). The number of patients with a long system diagnostic delay was and those with a long patient diagnostic delay were 125 patients (32.55%; *p* < 0.001) and 105 patients (27.34%; *p* < 0.001), respectively (Table 3).

**Table 2:** Distribution of the different diagnostic delays of pulmonary tuberculosis cases diagnosed in the central region, in Burkina Faso, 2018

	Mean (days)	Median (days)	IQR	Minimum (days)	Maximum (days)
Patient diagnostic delay	50.92	21	7–52	1	720
System diagnostic delay	25.03	7	4–23.5	2	980
Total diagnostic time	75.90	37	21–89	5	1100

**Table 3:** Distribution of cases of pulmonary tuberculosis according to the modalities of the different diagnostic delays in the central region of Burkina Faso in 2018

Time to diagnosis	Effective	Percentage (%)	*p*
Patient diagnostic delay			*p* < 0.001
Short (≤ 30)	279	72.66	
Long (> 30)	105	27.34	
System diagnostic delay			*p* < 0.001
Short (≤ 15)	259	67.45	
Long (> 15)	125	32.55	
Total diagnostic time			*p* = 0.005
Short (≤ 45)	226	58.85	
Long (> 45)	158	41.25	

## Discussion

Our study, which included 384 patients, estimated the patient, system and total diagnostic delays to be, respectively, 21, 7 and 37 days. The mains limits of our study could include a possibility of recall bias, in particular with regard to the date of onset of the first symptoms, their duration and the date of the first seeking of care. Indeed, tuberculosis being a chronic disease with an insidious onset, it would make it difficult for some to remember exactly when symptoms started.

Moreover, the fact that the study did not take into account cases of extra-pulmonary tuberculosis and cases of negative bacilloscopic constitutes a limitation for generalization to all cases of TB.

However, the study is multi-center, bringing together 6 diagnostic and treatment centers from 5 health districts in the central region of the country. These diagnostic and treatment centers, in particular the center …. also constitute reference centers for the whole country. This confirms the possibility of generalization of the study to the whole country, see countries with the same health realities as Burkina Faso. These results constitute scientific evidence on which the National TB Control Program could draw on strategies to improve these delays, which are essential elements in the fight against TB.

These delays seem to decrease if we refer to the study carried out by Ouedraogo et al. [8] in 2006 in the country; this could reflect the results of the strategies implemented in the National Tuberculosis Control Program. These efforts should be maintained to achieve the objectives of sustainable development.

### Patient Diagnostic Delay

The median time to diagnosis due to the patient was 21 days. This period is short according to our definition but could still be improved to overcome this disease. For a disease whose most common symptom is cough, taking 21 days before consulting a health facility seems abnormal. The only explanation is that patients would resort to other means such as traditional medicine and self-medication, and seek care at a health facility only after failure or aggravation. This will delay the first contact with health services and by extension, therefore, lengthen the patient delay, and hence, the total diagnosis delay. In fact, traditional medicine is deeply rooted in populations; up to 35.15% of patients first consulted traditional medicine to access the referral health facility. Santos et al. [25] in Angola have shown an increase in the delay in diagnosis among patients who first turn to traditional medicine at the onset of signs. Ignorance helping, 11.2% of patients had self-medication to access the referral health facility. Awareness of the importance of seeking early care in health facilities and sensitization of traditional healers to refer patients to health facilities could be improved.

Patients seem to play a more important role in delaying TB diagnosis than the healthcare system. Shortening this period requires improving community communication on the symptoms of TB and the existence of structures and free care.

This delay was similar to the results of other studies in Africa, Ymer et al. [18] in Ethiopia in 2014 reported 21 days. Chahraoui et al. [26] in Algeria in 2016, Akrim et al. [11] in Morocco in 2014 reported 20 days. In Asia, Nathan et al. [22] in China in 2018 noted a median delay of 21 days. This could be explained by the fact that, such as these studies, our study was conducted in an urban setting, where patients could be more aware of the disease [41]

Our delay was less than that of 28–29 days reported in developed countries such as Saldana et al. [27] in the United Kingdom in 2013, Williams et al. [12] in Melbourne in 2017 and in most African countries, where this period varied from 28 to 61 days [4, 5, 10, 14, 16, 28–31]. These differences in delay between studies could be attributed to a disparity in their selection criteria; some studies included patients with all forms of TB. Indeed, in the literature, several studies have shown that patients with extra-pulmonary TB have experienced long delays compared to cases of pulmonary tuberculosis [18, 32].

However, our delay was greater than that of some studies, in particular that of Ndeikoundam et al. [33] in Chad in 2012; Tattevin et al. [34] in France in 2012; Kalan et al. [35] in Iran in 2018; Paramasivam et al. [20] in Kerala in 2017 which, respectively, found a median delay of 15; 14; 13 and 16 days. Two weeks is generally the recommended time for a cough patient to visit a health facility [36]. This may be due to the fact that, the majority of patients perceive their illness as a minor ailment and cough as common and often overlooked. However, the delay in the consultation has three essential consequences: the aggravation of the disease which can lead to death, the dissemination of the germ in the patient's environment and serious after-effects posing problems of management.

### System Diagnostic Delay

The median time to diagnosis due to the health system was 7 days in this study. This could be partly explained by the improvement of the sputum transport system in the laboratory network and the performance of health workers involved in the diagnosis and treatment of TB patients. The tuberculosis control center should continue efforts to reduce this delay to a maximum of 1–2 days in the era of GeneXpert® generalization. It was similar to that of Lusignani et al. [10] in 2013 in Angola (7 days), Bojovic et al. [37] in Montenegro in 2018 (7 days), Asefa et al. [14] in 2014 in Ethiopia (7 days). This could be explained by the common definition of diagnostic delay due to the system with these studies. On the other hand, it was shorter compared to the values of 30; 37; 45 and 77 days reported in Africa, respectively, by Chahraoui et al. [26] in Algeria in 2016, Ngangro et al. [33] in Chad in 2012, Osei et al. [5] in 2014 in Ghana; Buregyeya et al. [28] in Uganda in 2014. It was also lower than the values of studies carried out in Europe Tattevin et al. [34] in France in 2012; Saldana et al. [27] in the United Kingdom in 2013 who found a median delay due to the health system of 25 days and 39 days, respectively. This encouraging finding reflects the efficiency of the national tuberculosis control process. This process ranges from sputum collection and sputum examination to diagnosis and return of results to the health worker and transmission of information to the patient, followed by initiation of TB treatment among confirmed TB cases. Despite this positive result, rapid diagnostic tests should be used in the care process. Improving diagnostic services in lower level health facilities and increasing public awareness of the availability of such services closer to their place of residence could avoid unnecessary delays. However, an even shorter delay has been reported by Takarinda et al. [16] in Zimbabwe in 2015 and Xu et al. [24] in China in 2013 with a median delay of 2 days. This could be due to the high incidence of tuberculosis in those countries, where the health system would be more aware of tuberculosis.

### Total Diagnostic Delay

The median delay due to the health system (7 days) was significantly shorter than that due to the patient (21 days). Indeed, the delay due to the patient is a major contributor to the total diagnostic delay of this study. These data were similar to the results of recent studies carried out in Africa such as those of Osei et al. [5] in Ghana in 2014; Akrim et al. [11] in Morocco in 2014; that of Rabin et al. [38] in Georgia in 2013. This could be explained by the fact that the index of suspicion may be high in patients presenting late symptoms; health workers, therefore, quickly consider the diagnosis of tuberculosis. The fact that patient delay contributed significantly to total delay indicates that there are many opportunities to reduce total delay by intervening in the community. The median total diagnostic delay is 37 days with a mean of 75.9 days. The previous study carried out by Ouedraogo et al. [8] in 2006 in Burkina Faso noted an average delay of 120 days which is fortunately now reduced. A reduction of about a month and a half month in the total diagnostic delay is considered a great success in Burkina Faso. Despite the progress, we must remain vigilant and intensify the fight against the tuberculosis scourge, which continues to be a constant and growing threat due to the HIV pandemic and the fragile demographic and socio-economic balance of our country and the sub-region. This median total delay was similar to that found by Paramasivam et al. [20] in Kerala in 2017 (37 days), Mahato et al. [31] in Nepal in 2015 (39 days). This could be explained by the similarity of our study populations. However, it was lower than that found in the literature [15, 25, 28, 39–41]. This could be due to the fact that the majority of the delay studies were conducted in referral hospitals, where longer delays are expected depending on the patients, health care systems and, therefore, a long total diagnostic delay.

## Conclusion

This study revealed that the total diagnosis delay of pulmonary tuberculosis was long for almost half of the patients. On the other hand, patient delay is longer than system delay in patients with pulmonary TB. To reduce patient delay, it is crucial to increase public awareness of the signs and symptoms of the disease, to ensure the participation of traditional healers and to pay particular attention to disadvantaged groups. Health system delay can be further reduced through training, supervision and ongoing support of health workers as well as improved TB diagnostic services. Regulations prohibiting the dispensing of antibiotics, including anti-tuberculosis drugs, without a prescription should be enforced.

## Supplementary Information

Below is the link to the electronic supplementary material.

Supplementary file1 (DOCX 46 KB)

## Data Availability

Data are available on request to the author Yanogo Pauline Kiswendsida.

## Abbreviations

TB: Tuberculosis
HIV: Human immunodeficiency virus
AIDS: Acquired immunodeficiency syndrome
WHO: World Health Organization
DOTS: Directly observed treatment short-course
DTC: Diagnosis and treatment centers
STATA: Statistical software for data science
NTBCC: National tuberculosis control center
HSPC: Health and Social Promotion Centers
MCSA: Medical center with surgical antenna
UH: University hospital
MC: Medical center
