# Supplementary material for: Total, Patient and System Diagnostic Delays for Pulmonary Bacilliferous Tuberculosis in the Six Diagnostic and Treatment Centers in the Five Health Districts of the Central Region, Burkina Faso, 2018
Source: J Epidemiol Glob Health. 2022 Jan 3;12(1):124–32. doi: 10.1007/s44197-021-00027-z (PMC8907367; doi:10.1007/s44197-021-00027-z)
Supplement: Supplementary file 1 — Supplementary file1 (DOCX 46 KB) [file 44197_2021_27_MOESM1_ESM.docx]

+

**BURKINA FASO**

**Unity - Progress - Justice**

**MINISTRY OF HIGHER EDUCATION, SCIENTIFIC RESEARCH**

**AND INNOVATION**

**JOSEPH KI ZERBO UNIVERSITY**

# APPENDICES

**Collection sheet**

# I APPENDICES

**Collection sheet**

I. Identity

Identification number: / / diagnostic center: /

Age (years): <5 years / _ /; 5 –14 years / _ /; 15 – 24 years / _ /; 25 – 44 years / _/;

45 – 64 years / _ /; > 65 / _ /

Gender: 1 M / _ / 2 F / _ /

History of TB: 1 (Personal) / _ /; 2 (family) / _ /; 3 (Professional) / _ /, 4 / _ / no History of TB

Location: 1 Pulmonary / _ / 2 Extra pulmonary / _ /

Medical ATCD: 1. No / _ /

2. (chronic disease: diabetes; renal failure; cancer; hypertension) / _ /

3.HIV / system disease / _ /

4.treatment (corticosteroid therapy; chemotherapy; TNF inhibitors or anti-TNF = powerful anti-inflammatory drugs = all organ transplant treatments) / _ /

Housing: 1 (Home) / _ /; 2 (Community) / _ / (boarding school; prison; mines)

Profession: 1 (Unemployed + students + students other than medical / paramedics) / _ /

*“In the presence of the Masters of this School and of my dear classmates, I promise and I swear to be faithful to the laws of Honor and Probity in the practice of medicine. I will give my free care to the needy and never demand a salary above my work.*

*Admitted inside the houses, my eyes will not see what is happening there; my tongue will be silent on the secrets that will be entrusted to me and my condition will not serve to corrupt morals or to promote crimes.*

*Respectful and grateful to my Masters, I will return to their children the instruction I received from their fathers.*

*May men give me their esteem if I have stayed true to my promises. May I be shamed and despised by my colleagues if I fail. "*

2 workers / _ /

3.Health workers (medical students; paramedics; prison staff; TB researchers) / _ /

4.Other government officials / _ /

Residence: 1 (Urban) / _ /; 2 (Rural) / _ /

II. Reference location / patient journey

Region…………………………………………………………………………

District …………………………………………………………………………

Referral health facility (which requests the examination): 1 spontaneous consultation at the diagnostic center / _ /; 2 HSPC (primary level) / _ /; 3 Medical Center with Surgical Branch (secondary level)/ _ /; 4. UHC (tertiary level) / _ / 5 private structure / _ /.

Structure visited before the reference health facility: 1 (Health center) / _ /; 2 (Traditional) / _ /; 3 (Self-medication) / _ /

III. Clinical / paraclinical data

Symptoms of tuberculosis at the onset of the disease: 1 (cough) / _ /, 2 (fever) / _ /, 3 (weight loss) / _ /, 4 (night sweat) / _ /, 5 hemoptysis / _ / ; 6 chest pain / _ / sputum

HIV serology: 1 (positive) / _ / 2, (negative) / _ /

Diagnostic tool: 1 (microscopy) / _ / 2 (GeneXpert) / _ /.

IV. Data on diagnostic delays

Date of onset: days / _ /, months / _ /, year / _ /

Date of first health facility consultation: days / _ /, months / _ /, year / _ /

Date of patient’s consultation with the health service for diagnosis: days / _ /, month / _ /, year / _ /

Date diagnostic results obtained: days / _ /, months / _ /, year / _ /

Date of initiation of treatment: days / _ /, months / _ /, year / _ /
